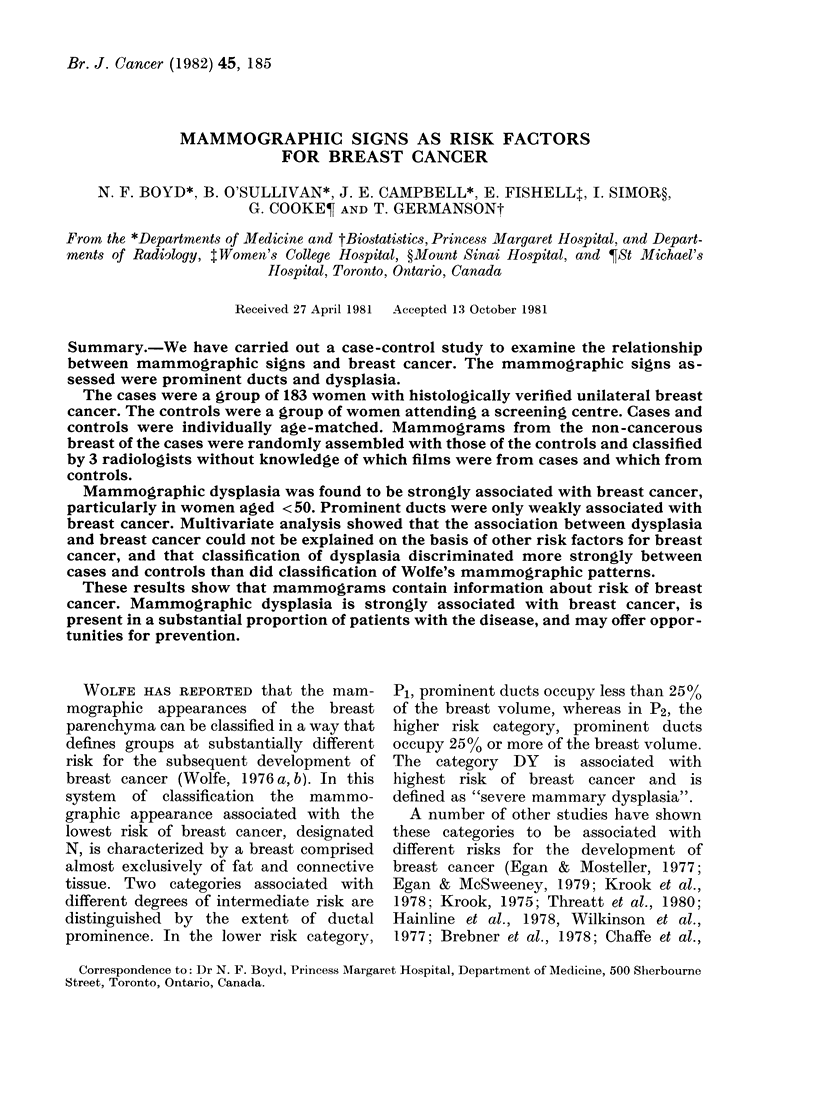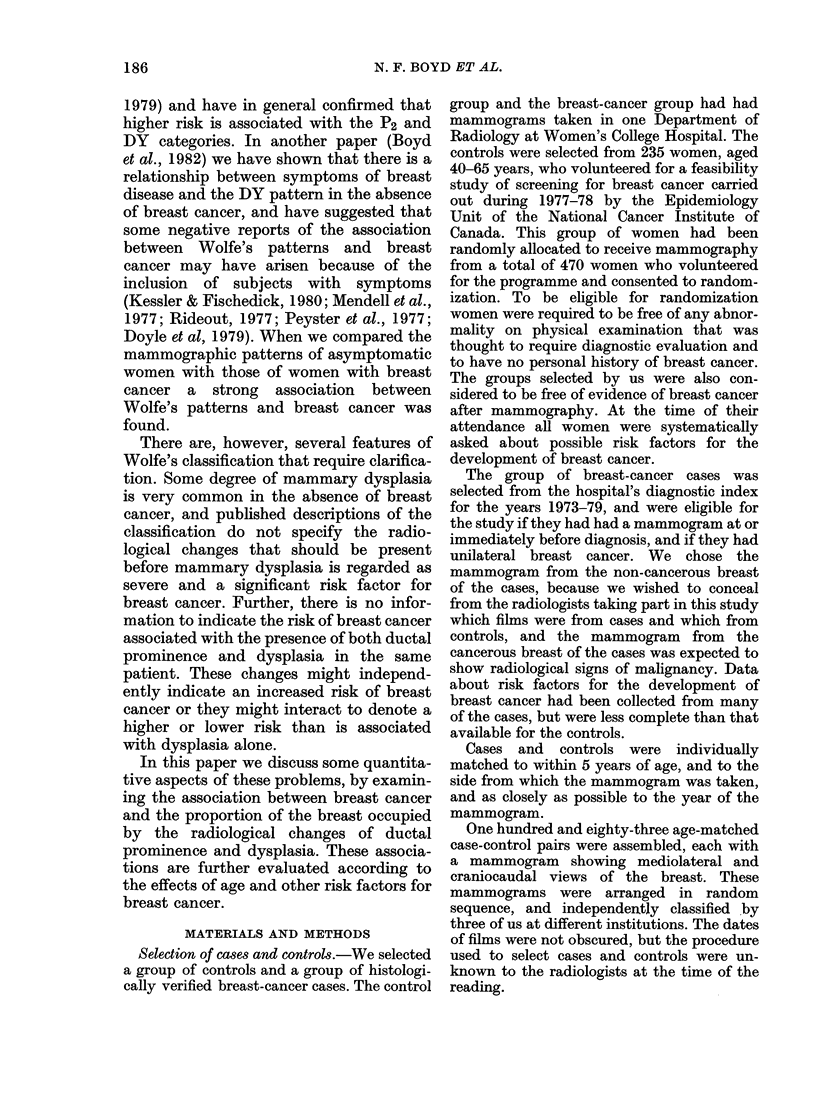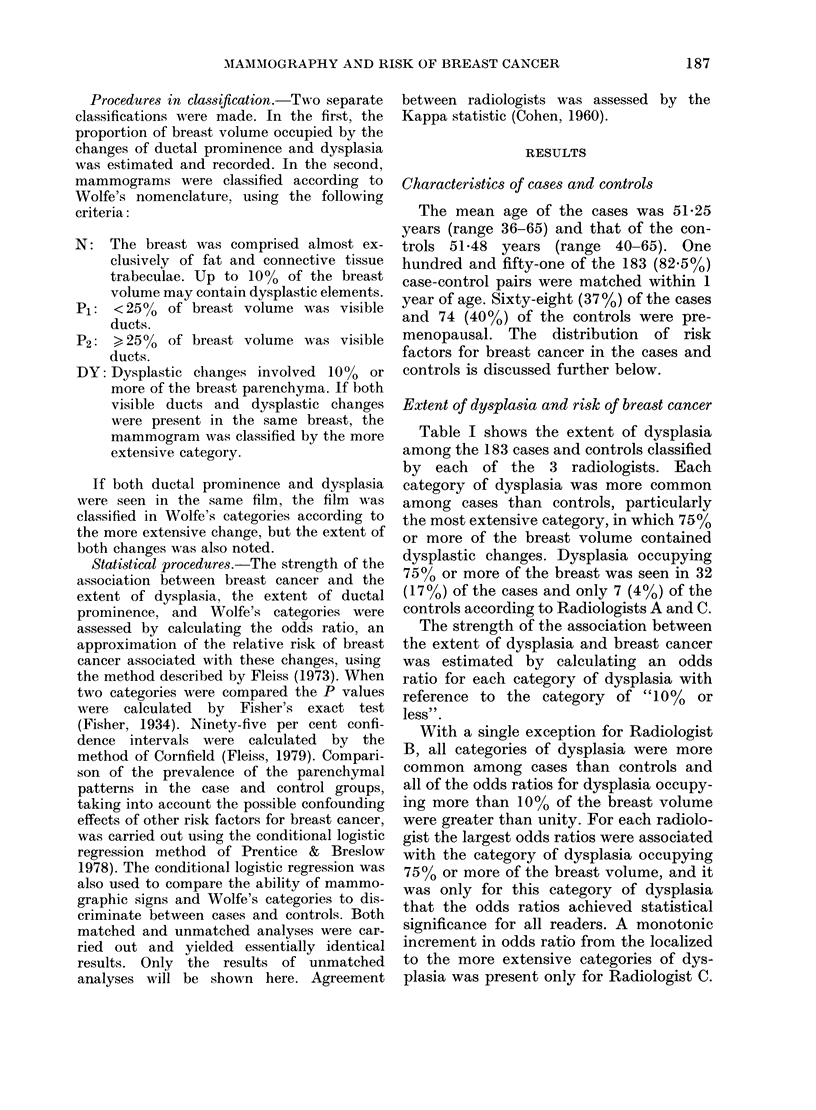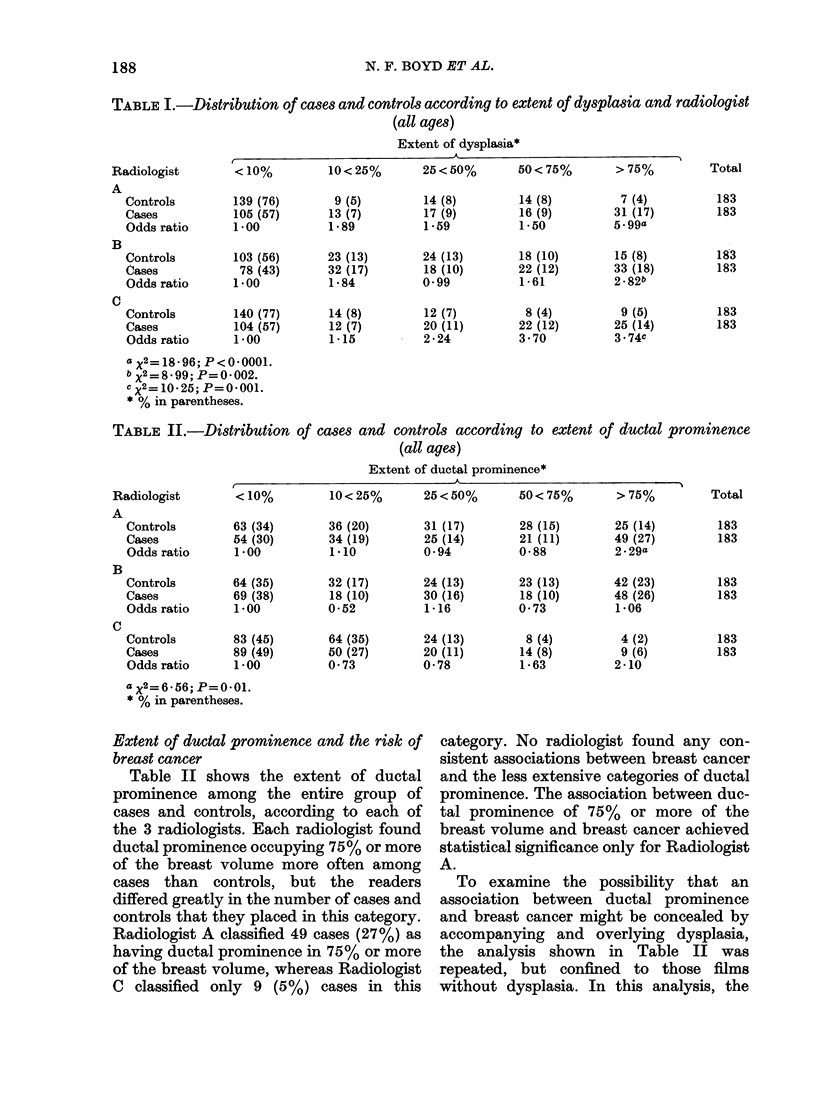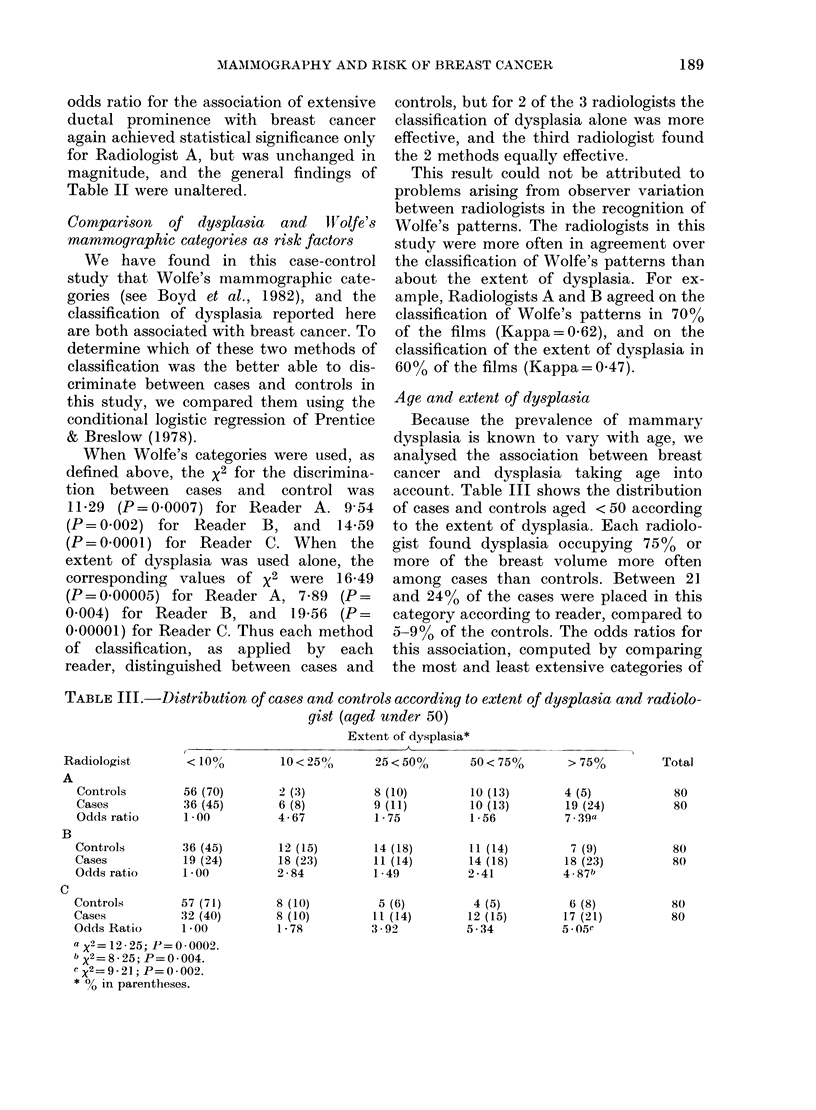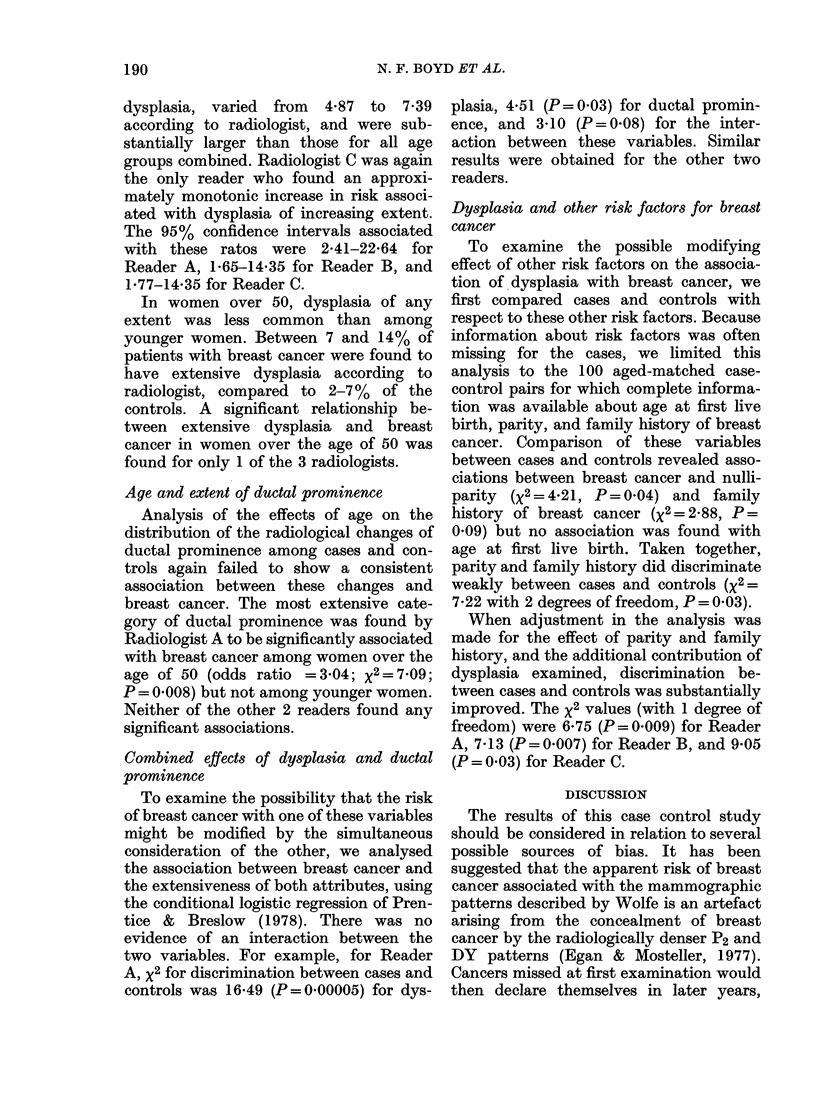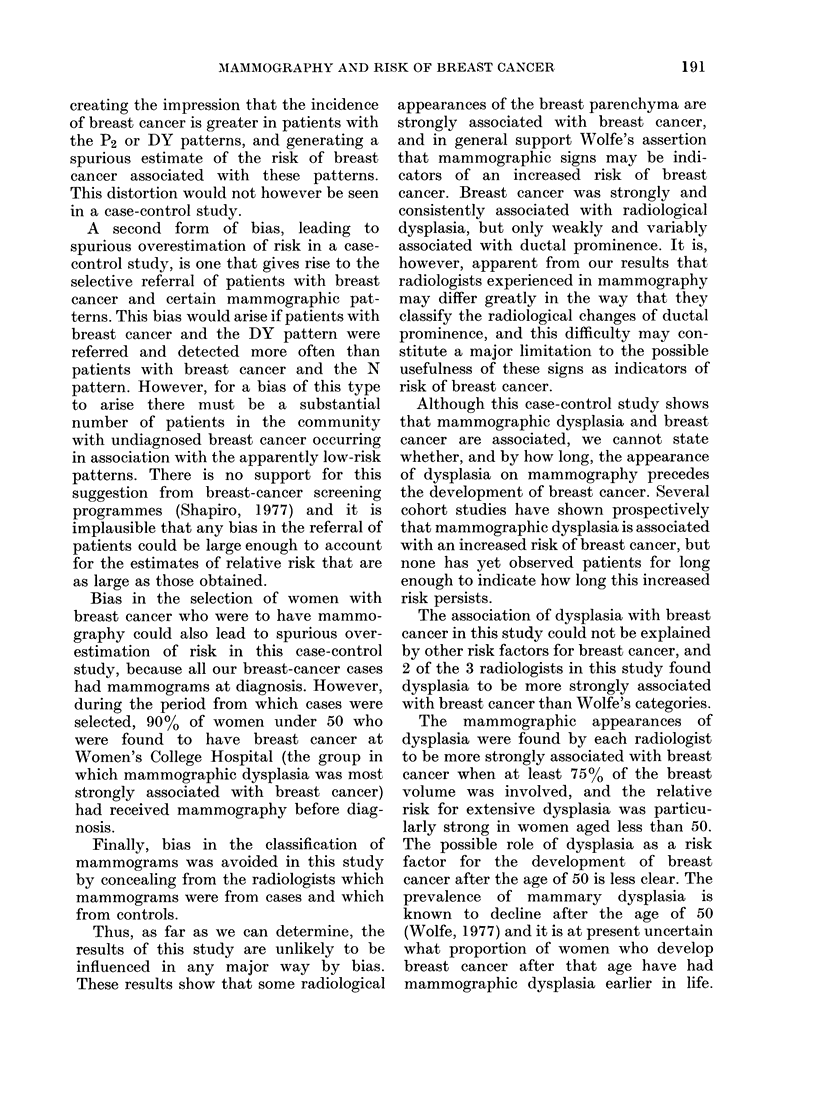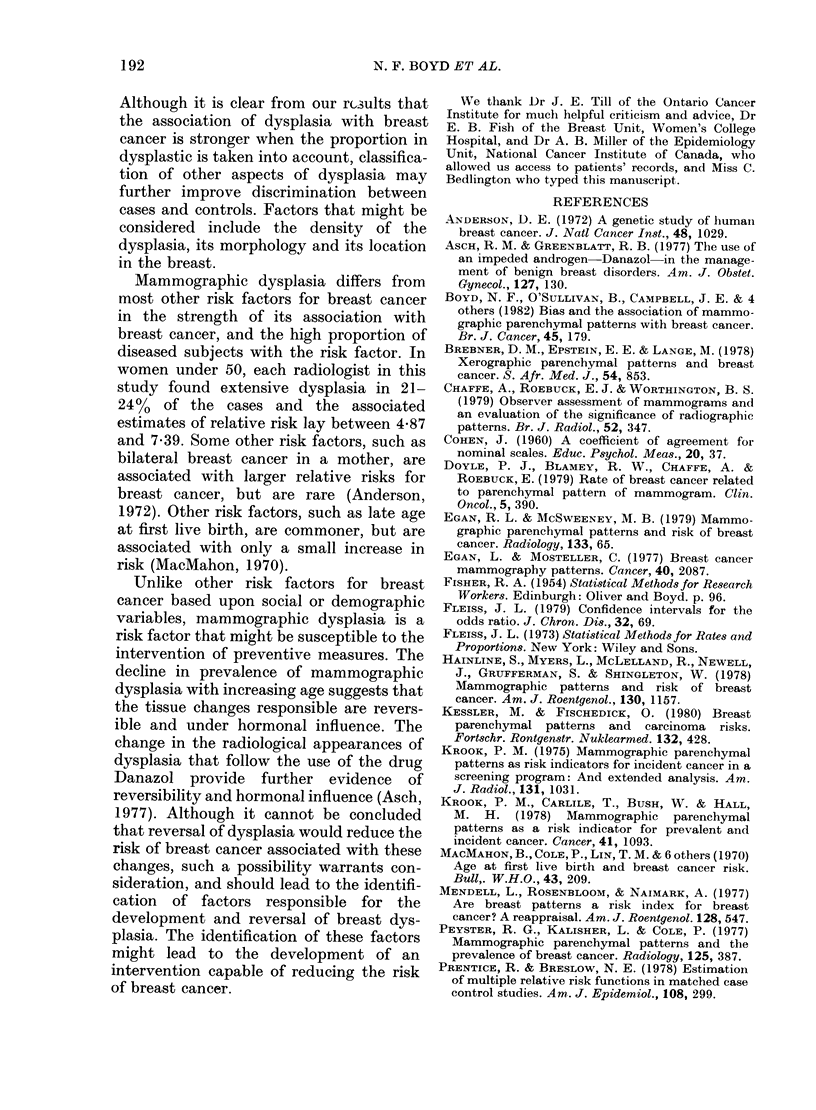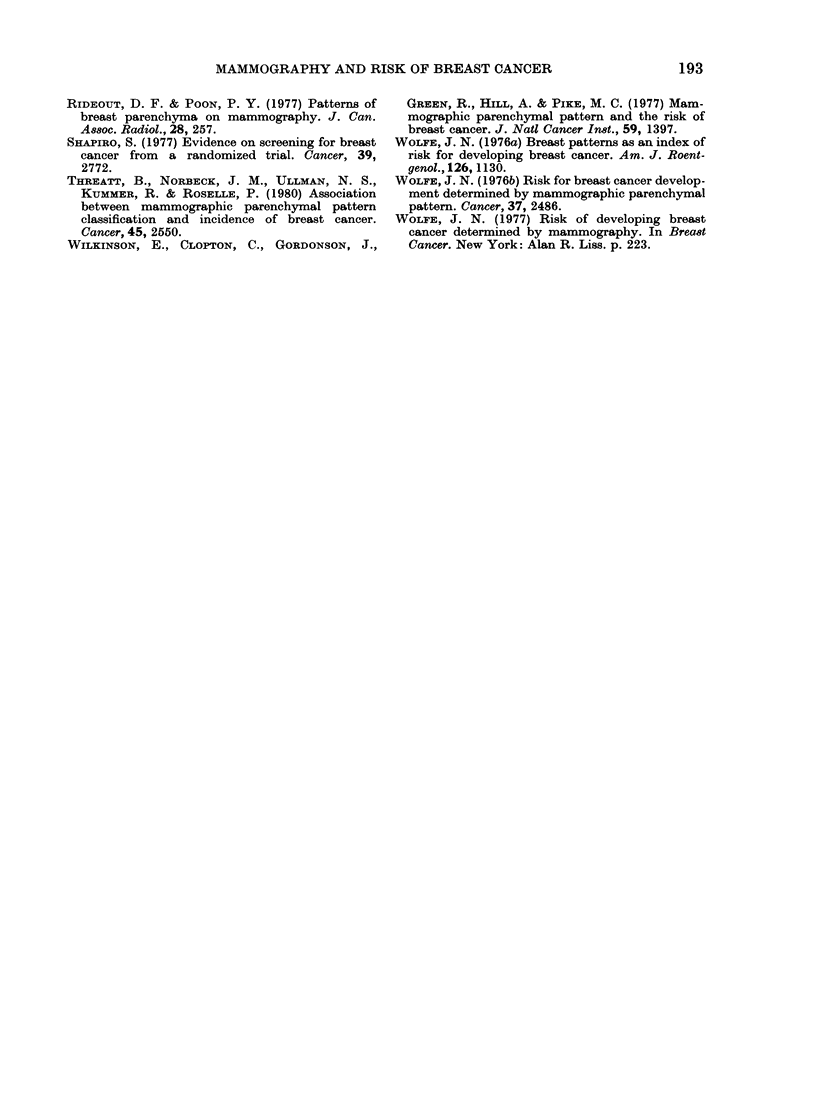# Mammographic signs as risk factors for breast cancer.

**DOI:** 10.1038/bjc.1982.32

**Published:** 1982-02

**Authors:** N. F. Boyd, B. O'Sullivan, J. E. Campbell, E. Fishell, I. Simor, G. Cooke, T. Germanson

## Abstract

We have carried out a case-control study to examine the relationship between mammographic signs and breast cancer. The mammographic signs assessed were prominent ducts and dysplasia. The cases were a group of 183 women with histologically verified unilateral breast cancer. The controls were a group of women attending a screening centre. Cases and controls were individually age-matched. Mammograms from the non-cancerous breast of the cases were randomly assembled with those of the controls and classified by 3 radiologists without knowledge of which films were from cases and which from controls. Mammographic dysplasia was found to be strongly associated with breast cancer, particularly in women aged less than 50. Prominent ducts were only weakly associated with breast cancer. Multivariate analysis showed that the association between dysplasia and breast cancer could not be explained on the basis of other risk factors for breast cancer, and that classification of dysplasia discriminated more strongly between cases and controls than did classification of Wolfe's mammographic patterns. These results show that mammograms contain information about risk of breast cancer. Mammographic dysplasia is strongly associated with breast cancer, is present in a substantial proportion of patients with the disease, and may offer opportunities for prevention.


*Br. J. Cancer* (1982) **45**, 185

### MAMMOGRAPHIC SIGNS AS RISK FACTORS FOR BREAST CANCER

N. F. BOYD*, B. O'SULLIVAN*, J. E. CAMPBELL*, E. FISHELL‡, I. SIMOR§, G. COOKE¶ AND T. GERMANSON†

*From the *Departments of Medicine and †Biostatistics, Princess Margaret Hospital, and Departments of Radiology, ‡Women's College Hospital, §Mount Sinai Hospital, and ¶St Michael's Hospital, Toronto, Ontario, Canada*

Received 27 April 1981 Accepted 13 October 1981

**Summary.—We have carried out a case-control study to examine the relationship between mammographic signs and breast cancer. The mammographic signs assessed were prominent ducts and dysplasia.**

**The cases were a group of 183 women with histologically verified unilateral breast cancer. The controls were a group of women attending a screening centre. Cases and controls were individually age-matched. Mammograms from the non-cancerous breast of the cases were randomly assembled with those of the controls and classified by 3 radiologists without knowledge of which films were from cases and which from controls.**

**Mammographic dysplasia was found to be strongly associated with breast cancer, particularly in women aged <50. Prominent ducts were only weakly associated with breast cancer. Multivariate analysis showed that the association between dysplasia and breast cancer could not be explained on the basis of other risk factors for breast cancer, and that classification of dysplasia discriminated more strongly between cases and controls than did classification of Wolfe's mammographic patterns.**

**These results show that mammograms contain information about risk of breast cancer. Mammographic dysplasia is strongly associated with breast cancer, is present in a substantial proportion of patients with the disease, and may offer opportunities for prevention.**

WOLFE HAS REPORTED that the mammographic appearances of the breast parenchyma can be classified in a way that defines groups at substantially different risk for the subsequent development of breast cancer (Wolfe, 1976*a*, *b*). In this system of classification the mammographic appearance associated with the lowest risk of breast cancer, designated N, is characterized by a breast comprised almost exclusively of fat and connective tissue. Two categories associated with different degrees of intermediate risk are distinguished by the extent of ductal prominence. In the lower risk category, $P_1$, prominent ducts occupy less than 25% of the breast volume, whereas in $P_2$, the higher risk category, prominent ducts occupy 25% or more of the breast volume. The category DY is associated with highest risk of breast cancer and is defined as "severe mammary dysplasia".

A number of other studies have shown these categories to be associated with different risks for the development of breast cancer (Egan & Mosteller, 1977; Egan & McSweeney, 1979; Krook *et al.*, 1978; Krook, 1975; Threatt *et al.*, 1980; Hainline *et al.*, 1978, Wilkinson *et al.*, 1977; Brebner *et al.*, 1978; Chaffe *et al.*,

Correspondence to: Dr N. F. Boyd, Princess Margaret Hospital, Department of Medicine, 500 Sherbourne Street, Toronto, Ontario, Canada.

1979) and have in general confirmed that higher risk is associated with the $P_2$ and DY categories. In another paper (Boyd *et al.*, 1982) we have shown that there is a relationship between symptoms of breast disease and the DY pattern in the absence of breast cancer, and have suggested that some negative reports of the association between Wolfe's patterns and breast cancer may have arisen because of the inclusion of subjects with symptoms (Kessler & Fischedick, 1980; Mendell *et al.*, 1977; Rideout, 1977; Peyster *et al.*, 1977; Doyle *et al*, 1979). When we compared the mammographic patterns of asymptomatic women with those of women with breast cancer a strong association between Wolfe's patterns and breast cancer was found.

There are, however, several features of Wolfe's classification that require clarification. Some degree of mammary dysplasia is very common in the absence of breast cancer, and published descriptions of the classification do not specify the radiological changes that should be present before mammary dysplasia is regarded as severe and a significant risk factor for breast cancer. Further, there is no information to indicate the risk of breast cancer associated with the presence of both ductal prominence and dysplasia in the same patient. These changes might independently indicate an increased risk of breast cancer or they might interact to denote a higher or lower risk than is associated with dysplasia alone.

In this paper we discuss some quantitative aspects of these problems, by examining the association between breast cancer and the proportion of the breast occupied by the radiological changes of ductal prominence and dysplasia. These associations are further evaluated according to the effects of age and other risk factors for breast cancer.

### MATERIALS AND METHODS

*Selection of cases and controls.*—We selected a group of controls and a group of histologically verified breast-cancer cases. The control group and the breast-cancer group had had mammograms taken in one Department of Radiology at Women's College Hospital. The controls were selected from 235 women, aged 40–65 years, who volunteered for a feasibility study of screening for breast cancer carried out during 1977–78 by the Epidemiology Unit of the National Cancer Institute of Canada. This group of women had been randomly allocated to receive mammography from a total of 470 women who volunteered for the programme and consented to randomization. To be eligible for randomization women were required to be free of any abnormality on physical examination that was thought to require diagnostic evaluation and to have no personal history of breast cancer. The groups selected by us were also considered to be free of evidence of breast cancer after mammography. At the time of their attendance all women were systematically asked about possible risk factors for the development of breast cancer.

The group of breast-cancer cases was selected from the hospital's diagnostic index for the years 1973–79, and were eligible for the study if they had had a mammogram at or immediately before diagnosis, and if they had unilateral breast cancer. We chose the mammogram from the non-cancerous breast of the cases, because we wished to conceal from the radiologists taking part in this study which films were from cases and which from controls, and the mammogram from the cancerous breast of the cases was expected to show radiological signs of malignancy. Data about risk factors for the development of breast cancer had been collected from many of the cases, but were less complete than that available for the controls.

Cases and controls were individually matched to within 5 years of age, and to the side from which the mammogram was taken, and as closely as possible to the year of the mammogram.

One hundred and eighty-three age-matched case-control pairs were assembled, each with a mammogram showing mediolateral and craniocaudal views of the breast. These mammograms were arranged in random sequence, and independently classified by three of us at different institutions. The dates of films were not obscured, but the procedure used to select cases and controls were unknown to the radiologists at the time of the reading.

*Procedures in classification.*—Two separate classifications were made. In the first, the proportion of breast volume occupied by the changes of ductal prominence and dysplasia was estimated and recorded. In the second, mammograms were classified according to Wolfe's nomenclature, using the following criteria:

N: The breast was comprised almost exclusively of fat and connective tissue trabeculae. Up to 10% of the breast volume may contain dysplastic elements.

$P_1$: <25% of breast volume was visible ducts.

$P_2$: ⩾25% of breast volume was visible ducts.

DY: Dysplastic changes involved 10% or more of the breast parenchyma. If both visible ducts and dysplastic changes were present in the same breast, the mammogram was classified by the more extensive category.

If both ductal prominence and dysplasia were seen in the same film, the film was classified in Wolfe's categories according to the more extensive change, but the extent of both changes was also noted.

*Statistical procedures.*—The strength of the association between breast cancer and the extent of dysplasia, the extent of ductal prominence, and Wolfe's categories were assessed by calculating the odds ratio, an approximation of the relative risk of breast cancer associated with these changes, using the method described by Fleiss (1973). When two categories were compared the *P* values were calculated by Fisher's exact test (Fisher, 1934). Ninety-five per cent confidence intervals were calculated by the method of Cornfield (Fleiss, 1979). Comparison of the prevalence of the parenchymal patterns in the case and control groups, taking into account the possible confounding effects of other risk factors for breast cancer, was carried out using the conditional logistic regression method of Prentice & Breslow 1978). The conditional logistic regression was also used to compare the ability of mammographic signs and Wolfe's categories to discriminate between cases and controls. Both matched and unmatched analyses were carried out and yielded essentially identical results. Only the results of unmatched analyses will be shown here. Agreement between radiologists was assessed by the Kappa statistic (Cohen, 1960).

### RESULTS

### *Characteristics of cases and controls*

The mean age of the cases was 51·25 years (range 36–65) and that of the controls 51·48 years (range 40–65). One hundred and fifty-one of the 183 (82·5%) case-control pairs were matched within 1 year of age. Sixty-eight (37%) of the cases and 74 (40%) of the controls were premenopausal. The distribution of risk factors for breast cancer in the cases and controls is discussed further below.

### *Extent of dysplasia and risk of breast cancer*

Table I shows the extent of dysplasia among the 183 cases and controls classified by each of the 3 radiologists. Each category of dysplasia was more common among cases than controls, particularly the most extensive category, in which 75% or more of the breast volume contained dysplastic changes. Dysplasia occupying 75% or more of the breast was seen in 32 (17%) of the cases and only 7 (4%) of the controls according to Radiologists A and C.

The strength of the association between the extent of dysplasia and breast cancer was estimated by calculating an odds ratio for each category of dysplasia with reference to the category of "10% or less".

With a single exception for Radiologist B, all categories of dysplasia were more common among cases than controls and all of the odds ratios for dysplasia occupying more than 10% of the breast volume were greater than unity. For each radiologist the largest odds ratios were associated with the category of dysplasia occupying 75% or more of the breast volume, and it was only for this category of dysplasia that the odds ratios achieved statistical significance for all readers. A monotonic increment in odds ratio from the localized to the more extensive categories of dysplasia was present only for Radiologist C.

TABLE I.—*Distribution of cases and controls according to extent of dysplasia and radiologist (all ages)*

| Radiologist | Extent of dysplasia* < 10% | 10 < 25% | 25 < 50% | 50 < 75% | > 75% | Total |
|---|---|---|---|---|---|---|
| A | | | | | | |
| Controls | 139 (76) | 9 (5) | 14 (8) | 14 (8) | 7 (4) | 183 |
| Cases | 105 (57) | 13 (7) | 17 (9) | 16 (9) | 31 (17) | 183 |
| Odds ratio | 1·00 | 1·89 | 1·59 | 1·50 | 5·99[a] | |
| B | | | | | | |
| Controls | 103 (56) | 23 (13) | 24 (13) | 18 (10) | 15 (8) | 183 |
| Cases | 78 (43) | 32 (17) | 18 (10) | 22 (12) | 33 (18) | 183 |
| Odds ratio | 1·00 | 1·84 | 0·99 | 1·61 | 2·82[b] | |
| C | | | | | | |
| Controls | 140 (77) | 14 (8) | 12 (7) | 8 (4) | 9 (5) | 183 |
| Cases | 104 (57) | 12 (7) | 20 (11) | 22 (12) | 25 (14) | 183 |
| Odds ratio | 1·00 | 1·15 | 2·24 | 3·70 | 3·74[c] | |

[a] $\chi^2 = 18\cdot96$; $P < 0\cdot0001$.

[b] $\chi^2 = 8\cdot99$; $P = 0\cdot002$.

[c] $\chi^2 = 10\cdot25$; $P = 0\cdot001$.

* % in parentheses.

TABLE II.—*Distribution of cases and controls according to extent of ductal prominence (all ages)*

| Radiologist | Extent of ductal prominence* < 10% | 10 < 25% | 25 < 50% | 50 < 75% | > 75% | Total |
|---|---|---|---|---|---|---|
| A | | | | | | |
| Controls | 63 (34) | 36 (20) | 31 (17) | 28 (15) | 25 (14) | 183 |
| Cases | 54 (30) | 34 (19) | 25 (14) | 21 (11) | 49 (27) | 183 |
| Odds ratio | 1·00 | 1·10 | 0·94 | 0·88 | 2·29[a] | |
| B | | | | | | |
| Controls | 64 (35) | 32 (17) | 24 (13) | 23 (13) | 42 (23) | 183 |
| Cases | 69 (38) | 18 (10) | 30 (16) | 18 (10) | 48 (26) | 183 |
| Odds ratio | 1·00 | 0·52 | 1·16 | 0·73 | 1·06 | |
| C | | | | | | |
| Controls | 83 (45) | 64 (35) | 24 (13) | 8 (4) | 4 (2) | 183 |
| Cases | 89 (49) | 50 (27) | 20 (11) | 14 (8) | 9 (6) | 183 |
| Odds ratio | 1·00 | 0·73 | 0·78 | 1·63 | 2·10 | |

[a] $\chi^2 = 6\cdot56$; $P = 0\cdot01$.

* % in parentheses.

### *Extent of ductal prominence and the risk of breast cancer*

Table II shows the extent of ductal prominence among the entire group of cases and controls, according to each of the 3 radiologists. Each radiologist found ductal prominence occupying 75% or more of the breast volume more often among cases than controls, but the readers differed greatly in the number of cases and controls that they placed in this category. Radiologist A classified 49 cases (27%) as having ductal prominence in 75% or more of the breast volume, whereas Radiologist C classified only 9 (5%) cases in this category. No radiologist found any consistent associations between breast cancer and the less extensive categories of ductal prominence. The association between ductal prominence of 75% or more of the breast volume and breast cancer achieved statistical significance only for Radiologist A.

To examine the possibility that an association between ductal prominence and breast cancer might be concealed by accompanying and overlying dysplasia, the analysis shown in Table II was repeated, but confined to those films without dysplasia. In this analysis, the odds ratio for the association of extensive ductal prominence with breast cancer again achieved statistical significance only for Radiologist A, but was unchanged in magnitude, and the general findings of Table II were unaltered.

*Comparison of dysplasia and Wolfe's mammographic categories as risk factors*

We have found in this case-control study that Wolfe's mammographic categories (see Boyd *et al.*, 1982), and the classification of dysplasia reported here are both associated with breast cancer. To determine which of these two methods of classification was the better able to discriminate between cases and controls in this study, we compared them using the conditional logistic regression of Prentice & Breslow (1978).

When Wolfe's categories were used, as defined above, the $\chi^2$ for the discrimination between cases and control was 11·29 ($P=0·0007$) for Reader A. 9·54 ($P=0·002$) for Reader B, and 14·59 ($P=0·0001$) for Reader C. When the extent of dysplasia was used alone, the corresponding values of $\chi^2$ were 16·49 ($P=0·00005$) for Reader A, 7·89 ($P=0·004$) for Reader B, and 19·56 ($P=0·00001$) for Reader C. Thus each method of classification, as applied by each reader, distinguished between cases and controls, but for 2 of the 3 radiologists the classification of dysplasia alone was more effective, and the third radiologist found the 2 methods equally effective.

This result could not be attributed to problems arising from observer variation between radiologists in the recognition of Wolfe's patterns. The radiologists in this study were more often in agreement over the classification of Wolfe's patterns than about the extent of dysplasia. For example, Radiologists A and B agreed on the classification of Wolfe's patterns in 70% of the films (Kappa = 0·62), and on the classification of the extent of dysplasia in 60% of the films (Kappa = 0·47).

*Age and extent of dysplasia*

Because the prevalence of mammary dysplasia is known to vary with age, we analysed the association between breast cancer and dysplasia taking age into account. Table III shows the distribution of cases and controls aged < 50 according to the extent of dysplasia. Each radiologist found dysplasia occupying 75% or more of the breast volume more often among cases than controls. Between 21 and 24% of the cases were placed in this category according to reader, compared to 5–9% of the controls. The odds ratios for this association, computed by comparing the most and least extensive categories of

TABLE III.—*Distribution of cases and controls according to extent of dysplasia and radiologist (aged under 50)*

| | Extent of dysplasia* | | | | | |
|---|---|---|---|---|---|---|
| Radiologist | < 10% | 10 < 25% | 25 < 50% | 50 < 75% | > 75% | Total |
| A | | | | | | |
| Controls | 56 (70) | 2 (3) | 8 (10) | 10 (13) | 4 (5) | 80 |
| Cases | 36 (45) | 6 (8) | 9 (11) | 10 (13) | 19 (24) | 80 |
| Odds ratio | 1·00 | 4·67 | 1·75 | 1·56 | 7·39[a] | |
| B | | | | | | |
| Controls | 36 (45) | 12 (15) | 14 (18) | 11 (14) | 7 (9) | 80 |
| Cases | 19 (24) | 18 (23) | 11 (14) | 14 (18) | 18 (23) | 80 |
| Odds ratio | 1·00 | 2·84 | 1·49 | 2·41 | 4·87[b] | |
| C | | | | | | |
| Controls | 57 (71) | 8 (10) | 5 (6) | 4 (5) | 6 (8) | 80 |
| Cases | 32 (40) | 8 (10) | 11 (14) | 12 (15) | 17 (21) | 80 |
| Odds Ratio | 1·00 | 1·78 | 3·92 | 5·34 | 5·05[c] | |

[a] $\chi^2 = 12·25$; $P = 0·0002$.

[b] $\chi^2 = 8·25$; $P = 0·004$.

[c] $\chi^2 = 9·21$; $P = 0·002$.

* % in parentheses.

dysplasia, varied from 4·87 to 7·39 according to radiologist, and were substantially larger than those for all age groups combined. Radiologist C was again the only reader who found an approximately monotonic increase in risk associated with dysplasia of increasing extent. The 95% confidence intervals associated with these ratos were 2·41–22·64 for Reader A, 1·65–14·35 for Reader B, and 1·77–14·35 for Reader C.

In women over 50, dysplasia of any extent was less common than among younger women. Between 7 and 14% of patients with breast cancer were found to have extensive dysplasia according to radiologist, compared to 2–7% of the controls. A significant relationship between extensive dysplasia and breast cancer in women over the age of 50 was found for only 1 of the 3 radiologists.

*Age and extent of ductal prominence*

Analysis of the effects of age on the distribution of the radiological changes of ductal prominence among cases and controls again failed to show a consistent association between these changes and breast cancer. The most extensive category of ductal prominence was found by Radiologist A to be significantly associated with breast cancer among women over the age of 50 (odds ratio $=3·04$; $\chi^2=7·09$; $P=0·008$) but not among younger women. Neither of the other 2 readers found any significant associations.

*Combined effects of dysplasia and ductal prominence*

To examine the possibility that the risk of breast cancer with one of these variables might be modified by the simultaneous consideration of the other, we analysed the association between breast cancer and the extensiveness of both attributes, using the conditional logistic regression of Prentice & Breslow (1978). There was no evidence of an interaction between the two variables. For example, for Reader A, $\chi^2$ for discrimination between cases and controls was 16·49 ($P=0·00005$) for dysplasia, 4·51 ($P=0·03$) for ductal prominence, and 3·10 ($P=0·08$) for the interaction between these variables. Similar results were obtained for the other two readers.

*Dysplasia and other risk factors for breast cancer*

To examine the possible modifying effect of other risk factors on the association of dysplasia with breast cancer, we first compared cases and controls with respect to these other risk factors. Because information about risk factors was often missing for the cases, we limited this analysis to the 100 aged-matched case-control pairs for which complete information was available about age at first live birth, parity, and family history of breast cancer. Comparison of these variables between cases and controls revealed associations between breast cancer and nulliparity ($\chi^2=4·21$, $P=0·04$) and family history of breast cancer ($\chi^2=2·88$, $P=0·09$) but no association was found with age at first live birth. Taken together, parity and family history did discriminate weakly between cases and controls ($\chi^2=7·22$ with 2 degrees of freedom, $P=0·03$).

When adjustment in the analysis was made for the effect of parity and family history, and the additional contribution of dysplasia examined, discrimination between cases and controls was substantially improved. The $\chi^2$ values (with 1 degree of freedom) were 6·75 ($P=0·009$) for Reader A, 7·13 ($P=0·007$) for Reader B, and 9·05 ($P=0·03$) for Reader C.

### DISCUSSION

The results of this case control study should be considered in relation to several possible sources of bias. It has been suggested that the apparent risk of breast cancer associated with the mammographic patterns described by Wolfe is an artefact arising from the concealment of breast cancer by the radiologically denser $P_2$ and DY patterns (Egan & Mosteller, 1977). Cancers missed at first examination would then declare themselves in later years, creating the impression that the incidence of breast cancer is greater in patients with the $P_2$ or DY patterns, and generating a spurious estimate of the risk of breast cancer associated with these patterns. This distortion would not however be seen in a case-control study.

A second form of bias, leading to spurious overestimation of risk in a case-control study, is one that gives rise to the selective referral of patients with breast cancer and certain mammographic patterns. This bias would arise if patients with breast cancer and the DY pattern were referred and detected more often than patients with breast cancer and the N pattern. However, for a bias of this type to arise there must be a substantial number of patients in the community with undiagnosed breast cancer occurring in association with the apparently low-risk patterns. There is no support for this suggestion from breast-cancer screening programmes (Shapiro, 1977) and it is implausible that any bias in the referral of patients could be large enough to account for the estimates of relative risk that are as large as those obtained.

Bias in the selection of women with breast cancer who were to have mammography could also lead to spurious overestimation of risk in this case-control study, because all our breast-cancer cases had mammograms at diagnosis. However, during the period from which cases were selected, 90% of women under 50 who were found to have breast cancer at Women's College Hospital (the group in which mammographic dysplasia was most strongly associated with breast cancer) had received mammography before diagnosis.

Finally, bias in the classification of mammograms was avoided in this study by concealing from the radiologists which mammograms were from cases and which from controls.

Thus, as far as we can determine, the results of this study are unlikely to be influenced in any major way by bias. These results show that some radiological appearances of the breast parenchyma are strongly associated with breast cancer, and in general support Wolfe's assertion that mammographic signs may be indicators of an increased risk of breast cancer. Breast cancer was strongly and consistently associated with radiological dysplasia, but only weakly and variably associated with ductal prominence. It is, however, apparent from our results that radiologists experienced in mammography may differ greatly in the way that they classify the radiological changes of ductal prominence, and this difficulty may constitute a major limitation to the possible usefulness of these signs as indicators of risk of breast cancer.

Although this case-control study shows that mammographic dysplasia and breast cancer are associated, we cannot state whether, and by how long, the appearance of dysplasia on mammography precedes the development of breast cancer. Several cohort studies have shown prospectively that mammographic dysplasia is associated with an increased risk of breast cancer, but none has yet observed patients for long enough to indicate how long this increased risk persists.

The association of dysplasia with breast cancer in this study could not be explained by other risk factors for breast cancer, and 2 of the 3 radiologists in this study found dysplasia to be more strongly associated with breast cancer than Wolfe's categories.

The mammographic appearances of dysplasia were found by each radiologist to be more strongly associated with breast cancer when at least 75% of the breast volume was involved, and the relative risk for extensive dysplasia was particularly strong in women aged less than 50. The possible role of dysplasia as a risk factor for the development of breast cancer after the age of 50 is less clear. The prevalence of mammary dysplasia is known to decline after the age of 50 (Wolfe, 1977) and it is at present uncertain what proportion of women who develop breast cancer after that age have had mammographic dysplasia earlier in life.

Although it is clear from our results that the association of dysplasia with breast cancer is stronger when the proportion in dysplastic is taken into account, classification of other aspects of dysplasia may further improve discrimination between cases and controls. Factors that might be considered include the density of the dysplasia, its morphology and its location in the breast.

Mammographic dysplasia differs from most other risk factors for breast cancer in the strength of its association with breast cancer, and the high proportion of diseased subjects with the risk factor. In women under 50, each radiologist in this study found extensive dysplasia in 21–24% of the cases and the associated estimates of relative risk lay between 4·87 and 7·39. Some other risk factors, such as bilateral breast cancer in a mother, are associated with larger relative risks for breast cancer, but are rare (Anderson, 1972). Other risk factors, such as late age at first live birth, are commoner, but are associated with only a small increase in risk (MacMahon, 1970).

Unlike other risk factors for breast cancer based upon social or demographic variables, mammographic dysplasia is a risk factor that might be susceptible to the intervention of preventive measures. The decline in prevalence of mammographic dysplasia with increasing age suggests that the tissue changes responsible are reversible and under hormonal influence. The change in the radiological appearances of dysplasia that follow the use of the drug Danazol provide further evidence of reversibility and hormonal influence (Asch, 1977). Although it cannot be concluded that reversal of dysplasia would reduce the risk of breast cancer associated with these changes, such a possibility warrants consideration, and should lead to the identification of factors responsible for the development and reversal of breast dysplasia. The identification of these factors might lead to the development of an intervention capable of reducing the risk of breast cancer.

We thank Dr J. E. Till of the Ontario Cancer Institute for much helpful criticism and advice, Dr E. B. Fish of the Breast Unit, Women's College Hospital, and Dr A. B. Miller of the Epidemiology Unit, National Cancer Institute of Canada, who allowed us access to patients' records, and Miss C. Bedlington who typed this manuscript.

### REFERENCES

ANDERSON, D. E. (1972) A genetic study of human breast cancer. *J. Natl Cancer Inst.*, **48**, 1029.

ASCH, R. M. & GREENBLATT, R. B. (1977) The use of an impeded androgen—Danazol—in the management of benign breast disorders. *Am. J. Obstet. Gynecol.*, **127**, 130.

BOYD, N. F., O'SULLIVAN, B., CAMPBELL, J. E. & 4 others (1982) Bias and the association of mammographic parenchymal patterns with breast cancer. *Br. J. Cancer*, **45**, 179.

BREBNER, D. M., EPSTEIN, E. E. & LANGE, M. (1978) Xerographic parenchymal patterns and breast cancer. *S. Afr. Med. J.*, **54**, 853.

CHAFFE, A., ROEBUCK, E. J. & WORTHINGTON, B. S. (1979) Observer assessment of mammograms and an evaluation of the significance of radiographic patterns. *Br. J. Radiol.*, **52**, 347.

COHEN, J. (1960) A coefficient of agreement for nominal scales. *Educ. Psychol. Meas.*, **20**, 37.

DOYLE, P. J., BLAMEY, R. W., CHAFFE, A. & ROEBUCK, E. (1979) Rate of breast cancer related to parenchymal pattern of mammogram. *Clin. Oncol.*, **5**, 390.

EGAN, R. L. & MCSWEENEY, M. B. (1979) Mammographic parenchymal patterns and risk of breast cancer. *Radiology*, **133**, 65.

EGAN, L. & MOSTELLER, C. (1977) Breast cancer mammography patterns. *Cancer*, **40**, 2087.

FISHER, R. A. (1954) *Statistical Methods for Research Workers*. Edinburgh: Oliver and Boyd. p. 96.

FLEISS, J. L. (1979) Confidence intervals for the odds ratio. *J. Chron. Dis.*, **32**, 69.

FLEISS, J. L. (1973) *Statistical Methods for Rates and Proportions*. New York: Wiley and Sons.

HAINLINE, S., MYERS, L., MCLELLAND, R., NEWELL, J., GRUFFERMAN, S. & SHINGLETON, W. (1978) Mammographic patterns and risk of breast cancer. *Am. J. Roentgenol.*, **130**, 1157.

KESSLER, M. & FISCHEDICK, O. (1980) Breast parenchymal patterns and carcinoma risks. *Fortschr. Rontgenstr. Nuklearmed.* **132**, 428.

KROOK, P. M. (1975) Mammographic parenchymal patterns as risk indicators for incident cancer in a screening program: And extended analysis. *Am. J. Radiol.*, **131**, 1031.

KROOK, P. M., CARLILE, T., BUSH, W. & HALL, M. H. (1978) Mammographic parenchymal patterns as a risk indicator for prevalent and incident cancer. *Cancer*, **41**, 1093.

MACMAHON, B., COLE, P., LIN, T. M. & 6 others (1970) Age at first live birth and breast cancer risk. *Bull,. W.H.O.*, **43**, 209.

MENDELL, L., ROSENBLOOM, & NAIMARK, A. (1977) Are breast patterns a risk index for breast cancer? A reappraisal. *Am. J. Roentgenol.* **128**, 547.

PEYSTER, R. G., KALISHER, L. & COLE, P. (1977) Mammographic parenchymal patterns and the prevalence of breast cancer. *Radiology*, **125**, 387.

PRENTICE, R. & BRESLOW, N. E. (1978) Estimation of multiple relative risk functions in matched case control studies. *Am. J. Epidemiol.*, **108**, 299.

RIDEOUT, D. F. & POON, P. Y. (1977) Patterns of breast parenchyma on mammography. *J. Can. Assoc. Radiol.*, **28**, 257.

SHAPIRO, S. (1977) Evidence on screening for breast cancer from a randomized trial. *Cancer*, **39**, 2772.

THREATT, B., NORBECK, J. M., ULLMAN, N. S., KUMMER, R. & ROSELLE, P. (1980) Association between mammographic parenchymal pattern classification and incidence of breast cancer. *Cancer*, **45**, 2550.

WILKINSON, E., CLOPTON, C., GORDONSON, J., GREEN, R., HILL, A. & PIKE, M. C. (1977) Mammographic parenchymal pattern and the risk of breast cancer. *J. Natl Cancer Inst.*, **59**, 1397.

WOLFE, J. N. (1976*a*) Breast patterns as an index of risk for developing breast cancer. *Am. J. Roentgenol.*, **126**, 1130.

WOLFE, J. N. (1976*b*) Risk for breast cancer development determined by mammographic parenchymal pattern. *Cancer*, **37**, 2486.

WOLFE, J. N. (1977) Risk of developing breast cancer determined by mammography. In *Breast Cancer*. New York: Alan R. Liss. p. 223.